# Objective Sleep Architecture Alterations and Sleep-Dependent Brain Clearance Dysfunction Across the Early Alzheimer’s Disease Continuum: A Systematic Review

**DOI:** 10.3390/jcm15166454

**Published:** 2026-08-20

**Authors:** Sonja Cabarkapa, Courtney Shelton, Philippe Faucie, Jérôme Murgier

**Affiliations:** 1 St. Vincent’s Hospital, Melbourne, VIC 3065, Australia; 2 Austin Health, Heidelberg, VIC 3084, Australia; 3 Maison de Santé, Cabinet du Dr Philippe Faucie, 4 Avenue des Pyrénées, 64260 Arudy, France; 4 Aguilera Private Clinic–Ramsay Santé, 21 Rue de l’Estagnas, 64200 Biarritz, France

**Keywords:** Alzheimer’s disease, mild cognitive impairment, brain clearance, glymphatic system, sleep, slow-wave sleep, sleep oscillations, polysomnography, electroencephalography, DTI-ALPS, perivascular spaces, BOLD-CSF coupling, cognitive decline

## Abstract

**Background:** Sleep-dependent glymphatic clearance has emerged as a potential mechanism linking sleep disruption with Alzheimer’s Disease (AD) pathology. However, the relationship between objectively measured sleep and glymphatic function across the AD continuum remains unclear. **Methods:** Four databases (PubMed, Embase, Cochrane Library, and PsycINFO) were systematically searched for studies assessing objective sleep metrics and glymphatic-related biomarkers or clearance measures in humans across the AD continuum. Following peer review of the search strategy, supplementary searches of PubMed and Embase using expanded glymphatic and sleep electrophysiology terminology were undertaken to maximize sensitivity. The final database searches identified 416 records. After removal of 72 duplicates, 344 records were screened, 64 reports underwent full-text assessment, and four studies met the inclusion criteria. **Results:** Four studies involving participants across the AD continuum were included. Objective sleep assessment was performed using polysomnography or electroencephalography, while brain clearance was evaluated using direct or surrogate imaging measures including diffusion tensor image analysis along the perivascular space (DTI-ALPS), perivascular space burden, blood oxygen level-dependent–cerebrospinal fluid (BOLD-CSF) coupling, or direct tracer-based clearance imaging. Across studies, better preserved slow-wave sleep, slow-wave activity, and sleep oscillatory coupling were generally associated with more favorable glymphatic function or glymphatic-related biomarkers. Conversely, disrupted sleep architecture, reduced sleep efficiency, and altered sleep oscillatory coupling were associated with impaired glymphatic clearance or glymphatic dysfunction. **Conclusions:** Current evidence suggests that objectively measured sleep architecture, particularly slow-wave sleep and sleep oscillatory dynamics, may be associated with biomarkers of brain clearance across the AD continuum. However, the available evidence remains preliminary, is predominantly cross-sectional, and relies largely on indirect measures of brain clearance. Larger longitudinal studies incorporating standardized sleep assessment and validated measures of cerebral clearance are required to clarify temporal relationships, establish causality, and determine whether sleep-targeted interventions influence brain clearance or disease progression. **Summary of findings:** Preliminary evidence suggests that preserved slow-wave sleep and sleep oscillatory activity are associated with more favorable biomarkers of brain clearance, whereas disrupted sleep architecture is associated with less favorable clearance-related measures.

## 1. Introduction 

Alzheimer’s disease (AD) is the leading cause of dementia and represents an increasing global health challenge, with the number of people living with dementia projected to reach 139 million by 2050 [1,2]. Increasing evidence suggests that sleep disturbances occur early in the AD continuum and may contribute to disease progression before the onset of overt cognitive impairment. Alterations in sleep architecture, including reduced slow-wave sleep (SWS) and rapid eye movement (REM) sleep and increased sleep fragmentation, have been associated with amyloid-β (Aβ) and tau accumulation, cognitive decline, and an increased risk of developing AD [3,4]. Experimental and clinical studies suggest that one mechanism linking sleep disruption with AD pathology may involve impairment of the brain’s waste clearance systems. During sleep, particularly SWS, cerebrospinal fluid (CSF)–interstitial fluid exchange is enhanced, facilitating the removal of metabolic waste products, including Aβ and tau, through glymphatic and related perivascular clearance pathways [5]. Sleep deprivation and disruption of normal sleep architecture have been associated with impaired clearance, increased protein accumulation, and altered glymphatic biomarkers in both animal models and humans [6].

Although the glymphatic system has emerged as a major focus of interest, direct assessment of glymphatic-related biomarkers in humans remains challenging. Consequently, recent human studies [7] have employed several imaging biomarkers of cerebral clearance, including diffusion tensor image analysis along the perivascular space (DTI-ALPS) [8], MRI-visible perivascular spaces (PVS) [9], blood oxygen level-dependent–cerebrospinal fluid (BOLD-CSF) coupling [10], and intrathecal contrast-enhanced MRI [11,12] to investigate sleep-dependent brain clearance mechanisms. Several reviews have examined sleep disturbances, glymphatic dysfunction, or AD pathology individually. However, no previous systematic review has specifically synthesized evidence relating objectively measured sleep architecture to direct or surrogate markers of glymphatic function and sleep-dependent brain clearance across the AD continuum.

Therefore, the aim of this systematic review was to synthesize current evidence examining associations between objective sleep measures and glymphatic-related biomarkers across the AD continuum. By focusing on objective assessments of both sleep and cerebral clearance, this review sought to determine whether alterations in sleep architecture are associated with impaired brain clearance mechanisms in individuals with Alzheimer’s disease and its prodromal stages.

## 2. Methods

The purpose of this study was to conduct a systematic review evaluating the association between objective sleep measures and glymphatic-related biomarkers across the early AD continuum. The review was prospectively registered with the International Prospective Register of Systematic Reviews (PROSPERO; registration no. CRD420261403666). The review was conducted and reported in accordance with the Preferred Reporting Items for Systematic Reviews and Meta-Analyses (PRISMA) 2020 Statement [13].

**Figure 1:**
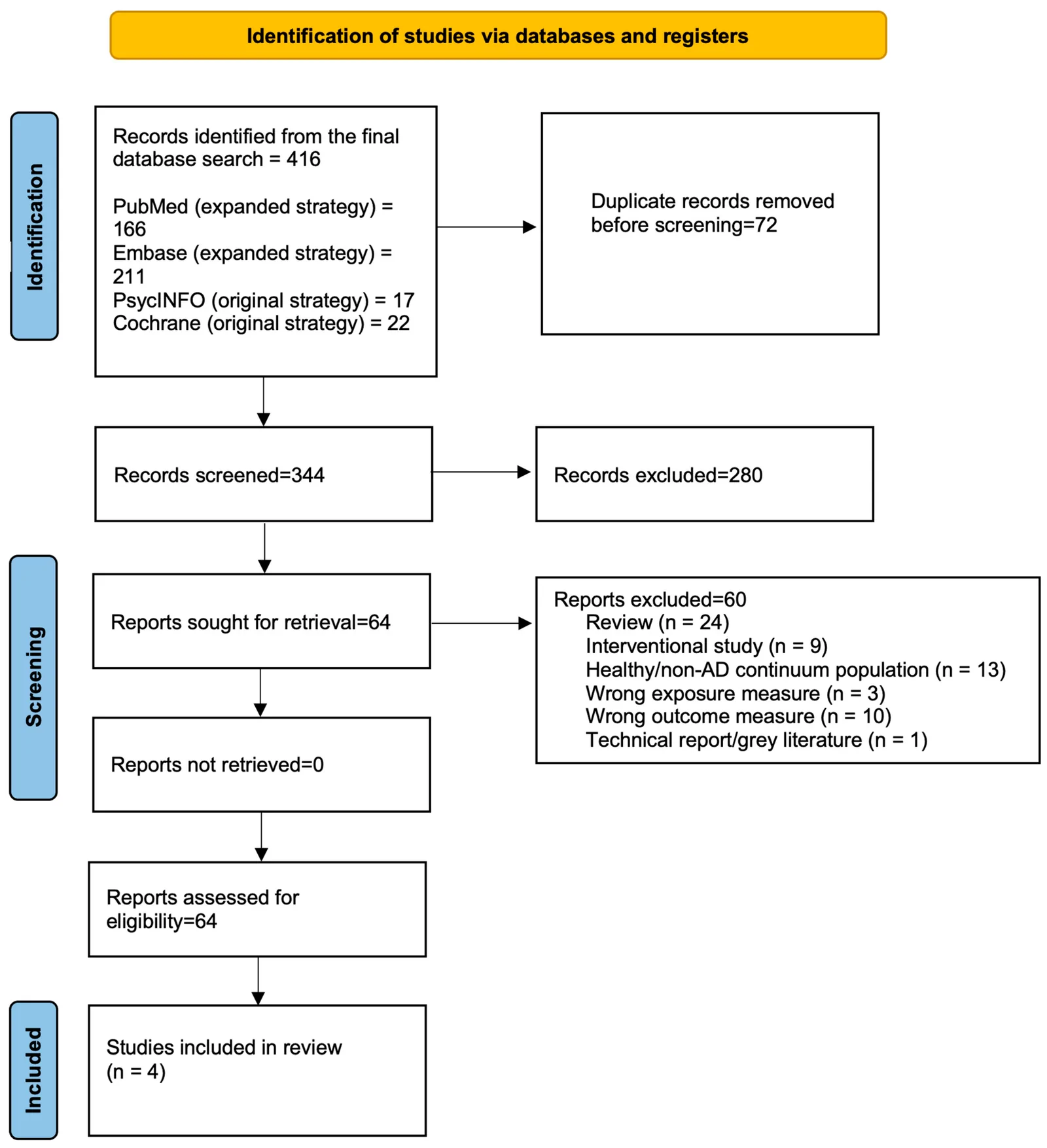
PRISMA 2020 flow diagram.

Search strategies for the four databases (PubMed, Embase, Cochrane Library, and PsycINFO) were developed by a psychiatry registrar in accordance with PRISMA 2020 recommendations and thereafter modified by a senior colleague (consultant psychiatrist with research experience) to ensure suitability. Following peer review of the initial search strategy, the PubMed and Embase searches were expanded using additional glymphatic, sleep electrophysiology, and preclinical AD terminology to improve sensitivity. The final systematic search comprised the expanded PubMed and Embase strategies together with the original PsycINFO and Cochrane Library searches. The flow diagram in Figure 1 provides details on the search strategy and the number of articles each database yielded, while search strategies are provided in Supplementary Tables S1 and S2. The four databases were searched on 9 June 2026 using combinations of terms related to sleep, glymphatic and brain clearance mechanisms, and AD. Search terms included sleep architecture, slow-wave sleep, rapid eye movement (REM), non-rapid eye movement (NREM), polysomnography, actigraphy, glymphatic, cerebrospinal fluid, amyloid, tau, perivascular spaces, AD, mild cognitive impairment, and subjective cognitive decline. Results were screened according to predefined inclusion and exclusion criteria. The supplementary search of two databases (PubMed and Embase) was performed on the 16 June 2026. 

Inclusion criteria for the qualitative synthesis were as follows:

1. Objective sleep metrics
2. And at least one of the following:
  - direct glymphatic imaging/measurement;
  - CSF dynamics;
  - amyloid/tau clearance-related biomarkers;
  - perivascular clearance markers;
  - glymphatic proxy measures.
3. Population:
  - Mild Cognitive Impairment (MCI);
  - early AD;
  - subjective cognitive decline;
  - cognitively normal older adults WITH AD-related biomarkers/risk/pathology.

Excluded studies

(1) not written in English;
(2) interventional studies;
(3) non-peer reviewed papers, proceedings, editorials, and reviews;
(4) the study population focused on healthy young adults, sleep studies unrelated to neurodegeneration and general insomnia populations without AD relevance.

### 2.1. Data Extraction

Data were independently extracted by two reviewers to identify the following variables: study author and year, country, study design, sample size, participant characteristics and position along the AD continuum, objective sleep-assessment method, sleep variables, glymphatic or cerebral-clearance measure, duration of follow-up, statistical approach, and principal findings. Records identified through the database searches were exported to Zotero (version 9.0.6; Corporation for Digital Scholarship, Vienna, VA, USA; https://www.zotero.org; accessed 15 July 2026) for duplicate removal. The deduplicated records were then imported into the Rayyan web application (Qatar Computing Research Institute, Doha, Qatar; https://www.rayyan.ai; accessed 15 July 2026) for independent title and abstract screening by two reviewers. Full-text articles deemed potentially eligible were assessed independently against the predefined inclusion and exclusion criteria. Disagreements were resolved through discussion and consensus, with consultation of a third reviewer when necessary. The third reviewer being a geriatrician with the highest degree of experience and knowledge on the topic of AD. 

### 2.2. Quality Analysis

The methodological quality and risk of bias of the included observational studies were independently assessed by two reviewers using the National Institutes of Health (NIH) Quality Assessment Tool for Observational Cohort and Cross-Sectional Studies [14]. The tool evaluates 14 methodological domains, including the clarity of the research question, study population, participation rate, participant selection, sample size justification, exposure and outcome assessment, temporal relationship, blinding of outcome assessors, follow-up, and statistical analyses.

As one included study was a case series, its methodological quality was assessed using the Joanna Briggs Institute (JBI) Critical Appraisal Checklist for Case Series [15] as demonstrated in Supplementary Table S3. This checklist evaluates key methodological domains, including clearly defined inclusion criteria, standardized measurement of the condition, valid methods for case identification, consecutive and complete inclusion of participants, detailed reporting of participant demographics and clinical information, appropriate outcome reporting, and the suitability of statistical analyses. 

Each NIH domain, as shown in Table 1, was rated as *Yes* (low risk of bias), *No* (high risk of bias), *Cannot determine*, *Not reported*, or *Not applicable* according to NIH guidance. Observational studies were subsequently classified as good, fair, or poor quality. The JBI checklist was used to provide a structured assessment of the methodological quality of the case series. Disagreements between reviewers were resolved through discussion until consensus was reached. The NIH tool does not prescribe numerical cut-offs for overall quality; ratings were therefore assigned by reviewer judgement based on the number and potential impact of unmet criteria.

The quality assessments of all included studies are demonstrated in Table 2. Three studies [16,17,18] were rated as fair quality, primarily due to well-defined populations, objective sleep and imaging assessments, and appropriate statistical analyses. However, no study reported a formal sample size calculation or blinded outcome assessment. Given several methodological limitations that were unlikely to invalidate the findings, they were considered fair. Particular consideration was given to participant selection, outcome assessment, control of confounding, and the appropriateness of statistical analyses.

Since Buongiorno et al. [19] was a case series rather than an observational study, methodological quality was assessed using the Joanna Briggs Institute (JBI) Critical Appraisal Checklist for Case Series instead of the NIH Quality Assessment Tool [15]. The study was generally well reported, with clear inclusion criteria, standardized diagnostic methods, detailed participant characteristics, comprehensive reporting of sleep and serial MRI findings, and documentation of adverse events. However, the study did not report whether participants were recruited consecutively or represented complete case inclusion, resulting in an “Unclear” rating for these domains. Furthermore, as an uncontrolled case series involving only four participants, the study remains inherently limited by selection bias, the absence of a comparator group, and limited generalizability despite the overall completeness of reporting.

Given the substantial heterogeneity in sleep metrics, glymphatic measures, participant characteristics, and study designs, a quantitative meta-analysis was not undertaken. Furthermore, because only four heterogeneous studies met the inclusion criteria, formal assessment of publication bias was not feasible. Likewise, the certainty of the body of evidence was not formally assessed using the GRADE approach because of the small number of studies and the absence of quantitative synthesis. Finally, studies were grouped according to the principal sleep metrics (slow-wave sleep, sleep oscillatory coupling, and sleep disturbance) and the corresponding glymphatic biomarkers.

## 3. Results

### 3.1. Study Characteristics 

Four studies [16,17,18,19] involving 397 participants met the inclusion criteria, including 267 participants across the AD continuum and 130 cognitively normal controls. Alzheimer’s disease continuum populations included biomarker-confirmed or clinically diagnosed AD, mild cognitive impairment due to AD, and biomarker-confirmed cognitive impairment consistent with AD. Two studies were conducted in China and two in Spain. Three were observational studies (two cross-sectional and one prospective cohort), while one was a case series. Sample sizes ranged from 4 to 260 participants. All studies employed objective sleep assessment using overnight polysomnography, with one study additionally analyzing electroencephalographic (EEG) oscillatory coupling. Brain clearance was evaluated using diffusion tensor image analysis along the perivascular space (DTI-ALPS; *n* = 2), blood oxygen level-dependent–cerebrospinal fluid (BOLD-CSF) coupling (*n* = 1), MRI-visible perivascular spaces (*n* = 1), locus coeruleus MRI (*n* = 1), and direct tracer-based MRI (*n* = 1). Study characteristics are summarized in Table 3. Due to substantial heterogeneity in sleep metrics, glymphatic measures, participant characteristics, and study design, a quantitative meta-analysis was not undertaken. Furthermore, since only four heterogeneous studies met the inclusion criteria, formal assessment of publication bias was not feasible. 

### 3.2. Relationship Between Slow-Wave Sleep and Brain-Clearance Biomarkers

Across studies, markers of deep NREM sleep were associated with several surrogate biomarkers of brain clearance. Falgàs et al. [18] reported that higher locus coeruleus integrity was associated with greater slow-wave activity (SWA) and slow oscillation (SO) power in participants with MCI due to AD. In the same study, greater basal ganglia perivascular space burden was associated with lower slow-wave spectral power, suggesting a relationship between impaired perivascular clearance pathways and disruption of restorative sleep [18].

Similarly, Liu et al. [16] reported that higher DTI-ALPS indices were associated with greater delta power and SWA in participants with AD. Lower DTI-ALPS values were associated with disrupted sleep oscillatory dynamics, suggesting that preserved slow-wave sleep physiology may be related to more favorable surrogate markers of brain clearance [16].

Although these studies used different imaging approaches and assessed distinct biological constructs, they consistently demonstrated associations between objective measures of slow-wave sleep and surrogate biomarkers related to cerebral clearance.

### 3.3. Sleep Oscillatory Coupling and Glymphatic Markers

One recent study examined the relationship between sleep oscillatory coupling and multiple surrogate measures of brain clearance [16]. Compared with cognitively normal controls, participants with AD demonstrated lower DTI-ALPS indices, reduced blood oxygen level-dependent–cerebrospinal fluid (BOLD-CSF) coupling, and impaired slow oscillation–spindle coupling. Lower DTI-ALPS values were associated with greater slow oscillation–spindle misalignment, whereas reduced BOLD-CSF coupling was associated with altered slow oscillation–theta coupling. In addition, combined sleep and clearance-related measures were associated with subsequent cognitive decline during follow-up [16].

These findings extend beyond traditional sleep-stage measures and suggest that coordinated sleep oscillations may be linked to glymphatic clearance mechanisms, consistent with experimental evidence demonstrating coupling between cortical slow waves, haemodynamic activity, and cerebrospinal fluid oscillations during NREM sleep [10] and with studies showing that age-related disruption of slow oscillation–spindle synchrony is associated with impaired memory consolidation and AD pathology [20,21]. However, because DTI-ALPS and BOLD-CSF coupling represent surrogate rather than direct measures of glymphatic function, these associations should be interpreted cautiously.

### 3.4. Direct Tracer-Based Assessment of Brain Clearance

Direct evidence of impaired brain clearance was provided by Buongiorno et al. [19], who combined overnight polysomnography (PSG) with serial MRI imaging following intrathecal gadobutrol administration in four individuals with cognitive impairment and biomarker evidence of AD pathology. Despite limited subjective sleep complaints, polysomnography demonstrated substantial sleep abnormalities, including reduced sleep efficiency, reduced N3 sleep, and obstructive sleep apnea (OSA) [19]. OSA is characterized by intermittent hypoxia and sleep fragmentation, both of which may impair glymphatic clearance [22,23]. Participants who completed the imaging protocol showed delayed and persistent tracer retention within cortical and white matter regions up to 48 h after administration, suggesting impaired brain clearance [19]. Although limited by the very small sample size and case series design, this study was the only included investigation to directly evaluate molecular brain clearance rather than relying on surrogate imaging biomarkers. 

### 3.5. Overall Findings

All four included studies [16,17,18,19] reported associations between objectively measured sleep characteristics and either direct or surrogate markers of brain clearance (Table 4 and the summarized version; Supplementary Table S4). The most consistent observations involved measures of deep NREM sleep, including slow-wave activity, slow-oscillation power, and sleep oscillatory coupling, which were generally associated with more favorable imaging biomarkers related to cerebral clearance. Conversely, reduced sleep efficiency, disrupted sleep architecture, and altered sleep oscillatory coupling were associated with less favorable clearance-related measures.

Interpretation of these findings should consider the substantial methodological heterogeneity across studies. Most investigations relied on indirect imaging biomarkers, including DTI-ALPS, BOLD-CSF coupling, MRI-visible perivascular spaces, and locus coeruleus integrity, whereas only one small case series directly assessed tracer-based brain clearance. Consequently, although the available evidence suggests an association between objective sleep measures and biomarkers of brain clearance across the AD continuum, the findings remain preliminary and do not establish causality.

## 4. Discussion

### 4.1. Principal Findings

This systematic review examined the relationship between objectively measured sleep and glymphatic function across the AD continuum. Despite methodological heterogeneity, the included studies generally reported similar associations: sleep characteristics associated with restorative NREM sleep were linked to better glymphatic function, whereas sleep disruption was associated with impaired glymphatic clearance.

The most frequently reported associations involved slow-wave sleep. Measures of slow-wave activity, slow oscillation power, and delta activity were positively associated with DTI-ALPS indices and inversely associated with markers of perivascular pathology. These findings are consistent with accumulating experimental and neuroimaging evidence suggesting that slow-wave sleep represents the physiological state most conducive to glymphatic transport and metabolic waste clearance [10,24,25]. 

Although previous narrative reviews have proposed a mechanistic relationship between sleep disruption, glymphatic dysfunction, and AD [3,4,25], the present review extends this literature by systematically synthesizing studies using objective sleep assessment together with human glymphatic biomarkers across the AD continuum. The consistency of the observed associations across several complementary imaging modalities provides preliminary support for the biological plausibility of sleep-dependent brain clearance mechanisms in humans.

### 4.2. Potential Mechanisms

Various biological mechanisms may explain the observed relationship between sleep and glymphatic function. Experimental studies have demonstrated that slow-wave sleep is accompanied by synchronized neuronal activity, changes in extracellular space volume, and enhanced cerebrospinal fluid movement, all of which may facilitate the clearance of metabolic waste products including amyloid-β and tau [22]. More recently, Fultz et al. [10] demonstrated that slow oscillations during NREM sleep are tightly coupled with large cerebrospinal fluid oscillations, providing physiological evidence linking sleep electrophysiology with brain clearance mechanisms [10]. These observations are supported by animal studies demonstrating increased glymphatic transport during natural sleep compared with wakefulness [22,24].

The observed associations between sleep oscillatory coupling and glymphatic markers further suggest that not only sleep quantity but also the temporal organization of sleep-related neural activity may be important for efficient brain clearance. Coordinated interactions between slow oscillations and sleep spindles are thought to facilitate systems-level memory consolidation and have been implicated in amyloid-β and tau homeostasis. Consequently, disruption of slow oscillation–spindle coupling may represent an additional pathway through which sleep dysfunction contributes to glymphatic impairment and AD pathology.

These findings are biologically plausible given experimental evidence demonstrating that large-amplitude cortical slow waves are temporally coupled with cerebrospinal fluid oscillations during NREM sleep, thereby providing a potential physiological mechanism linking sleep microarchitecture with glymphatic transport [10]. Furthermore, age-related disruption of slow oscillation–spindle coupling has previously been associated with impaired memory consolidation and increased AD pathology [20,21], suggesting that alterations in sleep microarchitecture may contribute to neurodegeneration through multiple complementary mechanisms.

Although excluded from the formal synthesis because it did not include participants within the AD continuum, Roy et al. [23] reported lower DTI-ALPS values with increasing OSA severity. These findings provide contextual support for a relationship between sleep disruption and brain-clearance biomarkers in an established AD risk population but should not be interpreted as evidence from the AD continuum itself.

### 4.3. Implications for Alzheimer’s Disease

Experimental sleep deprivation increases interstitial amyloid-β concentrations, whereas chronic sleep disruption accelerates amyloid deposition in animal models [26]. Conversely, amyloid and tau accumulation progressively disrupt sleep-regulating neural circuits, particularly within the locus coeruleus and other ascending arousal pathways, contributing to further deterioration of sleep architecture [3,20]. The observed relationships between sleep architecture, sleep oscillatory coupling, and glymphatic function support the hypothesis that sleep disruption and impaired brain clearance are closely linked in the pathophysiology of AD. Rather than representing independent phenomena, these processes may form part of a cycle that begins during the preclinical stages of the disease.

Reduced slow-wave sleep may impair the clearance of amyloid-β and tau, promoting their progressive accumulation. In turn, increasing amyloid and tau pathology disrupt sleep-regulating neural circuits—including the locus coeruleus, basal forebrain, and other ascending arousal networks—leading to further deterioration of sleep architecture and potentially accelerating neurodegeneration [3,20,26]. These findings support growing interest in sleep as a modifiable therapeutic target during the preclinical stages of AD. Sleep quality optimization, OSA treatment, and interventions aimed at enhancing slow-wave sleep may represent strategies for preserving brain clearance mechanisms, although clinical efficacy remains to be established.

### 4.4. Strengths

This review has several strengths. To our knowledge, it is the first systematic review specifically integrating evidence relating objective sleep architecture to glymphatic function across the AD continuum. Only studies employing objective sleep measurements (PSG or EEG) were included, thereby avoiding the limitations associated with subjective sleep questionnaires. Furthermore, multiple direct and surrogate biomarkers of brain clearance, including DTI-ALPS, BOLD-CSF coupling, MRI-visible PVS, and intrathecal contrast-enhanced MRI, were evaluated, providing a comprehensive overview of current human imaging approaches.

### 4.5. Limitations

Only one included study directly measured molecular brain clearance; the remaining studies relied on surrogate imaging biomarkers of uncertain equivalence. Overall, the certainty of the available evidence remains low to moderate owing to the small number of studies, predominantly cross-sectional designs, modest sample sizes, and reliance on surrogate rather than direct measures of glymphatic function. Furthermore, given the predominantly cross-sectional evidence, reverse causation cannot be excluded, and shared confounding by age, vascular disease, OSA, sex, medication use, and other comorbidities may contribute to the observed associations.

### 4.6. Future Directions

Future research should prioritize longitudinal multimodal studies combining polysomnography with advanced glymphatic imaging to determine whether sleep-related alterations precede biomarker progression and cognitive decline. Standardization of DTI-ALPS acquisition and analysis protocols, validation of BOLD-CSF coupling as a biomarker, and wider application of intrathecal contrast-enhanced MRI will improve comparability across studies. Randomized clinical trials are required to determine whether interventions that improve sleep architecture influence biomarkers of brain clearance and whether any resulting changes translate into clinically meaningful cognitive outcomes.

## 5. Conclusions

This systematic review suggests that objective measures of sleep architecture, particularly slow-wave sleep and sleep oscillatory dynamics, are associated with direct and surrogate biomarkers of brain clearance across AD populations. However, the available evidence is limited by small sample sizes, predominantly cross-sectional designs, and reliance on indirect imaging biomarkers. Consequently, causality cannot be inferred. Larger longitudinal studies using standardized sleep assessment together with validated measures of brain clearance are required before the clinical significance of these associations can be established.

## Figures and Tables

**Table 1:** Quality assessment of included studies using the NIH Quality Assessment Tool for Observational Cohort and Cross-Sectional Studies.

NIH Criterion	Liu 2026 [16]	Zheng 2026 [17]	Falgàs 2026 [18]
1. Clearly stated research question	Y	Y	Y
2. Clearly defined study population	Y	Y	Y
3. Participation rate ≥50%	Y	Y	Y
4. Uniform selection criteria	Y	Y	Y
5. Sample size justification/power calculation	N	N	N
6. Exposure measured before outcome	NA	NA	NA
7. Sufficient timeframe to observe association	Y	NA	NA
8. Different exposure levels examined	N	Y	Y
9. Exposure measures clearly defined and reliable	Y	Y	Y
10. Exposure assessed more than once	N	N	N
11. Outcome measures clearly defined and reliable	Y	Y	Y
12. Outcome assessors blinded	NR	NR	NR
13. Loss to follow-up ≤20%	Y	NA	NA
14. Appropriate statistical analysis	Y	Y	Y
**Overall quality**	**Fair**	**Fair**	**Fair**

Abbreviations: Y = Yes (low risk of bias); N = No (high risk of bias); NR = Not reported; NA = Not applicable.

**Table 2:** Quality assessment of all four included studies.

Study	Tool	Overall Quality
Liu [16]	NIH	Fair
Zheng [17]	NIH	Fair
Falgàs [18]	NIH	Fair
Buongiorno [19]	JBI Case Series Checklist	Well reported; interpretation limited by small case series design

**Table 3:** Study characteristics.

Study	Country; Design	Population	AD Diagnostic Criteria	Objective Sleep Assessment	Brain-Clearance Assessment
Liu et al. [16]	China; prospective observational cohort	54 biomarker-supported AD, 21 cognitively normal controls (*n* = 75)	CSF biomarker-confirmed AD	PSG with sleep EEG; SO-theta and SO-spindle coupling	DTI-ALPS, BOLD-CSF coupling, choroid plexus volume, PVS burden (surrogate)
Zheng et al. [17]	China; cross-sectional case-control	162 clinically diagnosed AD, 98 healthy controls (*n* = 260)	Clinical diagnosis (NIA-AA criteria)	PSG (AHI, ODI, sleep stages, sleep efficiency, arousal index)	DTI-ALPS (surrogate)
Falgàs et al. [18]	Spain; cross-sectional cohort	30 MCI due to AD, 17 AD dementia, 11 healthy controls (*n* = 58)	Amyloid biomarker-confirmed AD (CSF/PET)	PSG (SWS duration, SWA, SO power, delta power)	LC MRI, PVS burden, CSF noradrenaline (surrogate/associated biomarkers)
Buongiorno et al. [19]	Spain; case series	Four participants with CSF biomarker-confirmed AD (*n* = 4)	CSF biomarker-confirmed AD	PSG	Serial gadobutrol-enhanced MRI (direct tracer clearance)

**Abbreviations:** AD, Alzheimer’s disease; AHI, apnea–hypopnoea index; BOLD-CSF, blood oxygen level-dependent–cerebrospinal fluid coupling; CSF, cerebrospinal fluid; DTI-ALPS, diffusion tensor image analysis along the perivascular space; EEG, electroencephalography; LC, locus coeruleus; MCI, mild cognitive impairment; MRI, magnetic resonance imaging; NIA-AA, National Institute on Aging–Alzheimer’s Association; ODI, oxygen desaturation index; OSA, obstructive sleep apnea; PET, positron emission tomography; PSG, polysomnography; PVS, perivascular spaces; REM, rapid eye movement; SO, slow oscillation; SWA, slow-wave activity; SWS, slow-wave sleep. **Note:** Brain-clearance assessments included both direct and surrogate measures. Direct assessment was performed using serial intrathecal gadobutrol-enhanced MRI to evaluate tracer clearance. Surrogate measures included diffusion tensor image analysis along the perivascular space (DTI-ALPS), blood oxygen level-dependent–cerebrospinal fluid (BOLD-CSF) coupling, MRI-visible perivascular space (PVS) burden, and neuromelanin-sensitive locus coeruleus (LC) MRI. These surrogate measures represent related but distinct imaging biomarkers and should not be interpreted as equivalent measures of glymphatic function.

**Table 4:** Quantitative synthesis of associations between objective sleep measures and direct or surrogate brain-clearance measures.

Study	Principal Quantitative Findings	Adjustment/Covariates	Interpretation
Liu et al. [16], 2026	Compared with cognitively normal controls, participants with AD had lower global DTI-ALPS (1.47 ± 0.15 vs. 1.60 ± 0.18; adjusted *p* = 0.029) and lower global BOLD-CSF coupling (0.14 ± 0.19 vs. 0.35 ± 0.16; adjusted *p* < 0.001). Global DTI-ALPS was associated with SO-spindle alignment (r = 0.338, FDR-adjusted *p* = 0.020), and global BOLD-CSF coupling with SO-theta alignment (r = 0.311, FDR-adjusted *p* = 0.018). In AD-only analysis, DTI-ALPS remained associated with SO-spindle alignment (r = 0.354, FDR-adjusted *p* = 0.048). Mediation analysis showed an indirect effect of DTI-ALPS on the relationship between SO-spindle misalignment and MMSE (β = 1.371, 95% bootstrap CI 0.063–3.116) and MoCA (β = 1.460, 95% CI 0.011–3.548). The combined MRI/sleep model predicted 2-year progression with AUC = 0.864 (95% CI 0.776–0.952).	Group comparisons adjusted for age, sex, education and TIV; DTI-ALPS additionally adjusted for WMH burden. Partial correlations controlled for age, sex, education, diagnostic group and TIV, with WMH additionally included for DTI-ALPS.	Multiple MRI-derived clearance proxies were associated with altered sleep oscillatory coupling, but these remain surrogate measures. The prospective prediction analysis strengthens temporal information, although it does not establish causality.
Zheng et al. [17], 2026	In AD, DTI-ALPS correlated inversely with AHI (ρ = −0.38, 95% CI −0.51 to −0.23; *p* < 0.001), ODI (ρ = −0.35, 95% CI −0.49 to −0.20; *p* < 0.001), N1 sleep (ρ = −0.41, 95% CI −0.54 to −0.26; *p* < 0.001) and arousal index (ρ = −0.33, 95% CI −0.47 to −0.18; *p* < 0.001), and positively with REM sleep (ρ = 0.29, 95% CI 0.14–0.43; *p* = 0.001). In adjusted AD-only regression, AHI remained associated with lower DTI-ALPS (standardized β = −0.37, *p* < 0.001); no corresponding association occurred in controls (β = 0.05, *p* = 0.634). The AHI × diagnostic-group interaction was significant (β = −0.41, *p* = 0.008). After adjustment for all measured sleep comorbidities, the association remained (β = −0.29, *p* = 0.006).	Primary multivariable model adjusted for age, sex, sleep efficiency and PLMI. Sensitivity analyses additionally considered insomnia, depression/anxiety, RBD, RLS, PLMS, AD severity and vascular burden (Fazekas score).	Greater OSA severity and sleep fragmentation were associated with lower DTI-ALPS specifically in clinically diagnosed AD. DTI-ALPS is an indirect diffusion-based proxy and is itself sensitive to age and white-matter/vascular factors.
Falgàs et al. [18], 2026	LC integrity correlated with SWA (r = 0.27, *p* = 0.043) and SO power (r = 0.29, *p* = 0.028). In adjusted models, LC integrity remained associated with SO power (β = 0.632, *p* = 0.001) and SWA (β = 0.532, *p* = 0.003). Significant LC × sex interactions were observed for SO (β = −1.481, *p* = 0.008) and SWA (β = −1.130, *p* = 0.039), indicating stronger associations in women. Basal ganglia PVS burden was inversely associated with SWA (β = −1.092, *p* = 0.034) and SO power (β = −1.125, *p* = 0.030). CSO-PVS burden was not associated with SO (β = −0.045, *p* = 0.699) or SWA (β = −0.006, *p* = 0.962).	LC models controlled for age, sex, disease stage (CDR), sleep medication and antidepressant use, and tested LC × sex interaction; additional models examined AHI. Reduced PVS models retained LC integrity, age, sex and LC × sex after removal of non-significant covariates.	Associations differed by specific sleep and imaging metric: LC integrity related mainly to SO/SWA, while basal-ganglia PVS burden showed inverse associations with these spectral measures. Neither LC MRI nor PVS burden constitutes a direct measure of clearance.
Buongiorno et al. [19], 2023	All four participants had low sleep efficiency (39.0–66.7%); AHI ranged from 8.4 to 40.9 events/h, with two participants meeting severe OSA thresholds (AHI 37.9 and 40.9). Of the two participants completing 48-h imaging, cortical tracer enrichment remained elevated: T001 increased from 57.6% at 5–6 h to 68.1% at 48 h, while T002 showed no reduction at 48 h. White-matter enrichment at 48 h was 19.0% and 22.6%, respectively.	No adjusted association estimates or inferential regression analyses were reported. This was a descriptive four-participant case series.	Serial intrathecal tracer MRI provides the most direct clearance assessment among the included studies, but the very small uncontrolled sample precludes estimation of an association effect or causal inference.

**Abbreviations:** AD, Alzheimer’s disease; AHI, apnea–hypopnoea index; BOLD-CSF, blood oxygen level-dependent–cerebrospinal fluid coupling; CSF, cerebrospinal fluid; DTI-ALPS, diffusion tensor image analysis along the perivascular space; EEG, electroencephalography; LC, locus coeruleus; MCI, mild cognitive impairment; MRI, magnetic resonance imaging; NIA-AA, National Institute on Aging–Alzheimer’s Association; ODI, oxygen desaturation index; OSA, obstructive sleep apnea; PET, positron emission tomography; PSG, polysomnography; PVS, perivascular spaces; REM, rapid eye movement; SO, slow oscillation; SWA, slow-wave activity; SWS, slow-wave sleep. **Note:** Brain-clearance assessments included both direct and surrogate measures. Direct assessment was performed using serial intrathecal gadobutrol-enhanced MRI to evaluate tracer clearance. Surrogate measures included diffusion tensor image analysis along the perivascular space (DTI-ALPS), blood oxygen level-dependent–cerebrospinal fluid (BOLD-CSF) coupling, MRI-visible perivascular space (PVS) burden, and neuromelanin-sensitive locus coeruleus (LC) MRI. These surrogate measures represent related but distinct imaging biomarkers and should not be interpreted as equivalent measures of glymphatic function.

## Data Availability

Extracted data supporting the findings of this review are available within the article and online through the information supplied in the Supplementary Materials.

## Supplementary Materials

The following supporting information can be downloaded at: https://www.mdpi.com/article/10.3390/jcm15166454/s1, Supplementary Table S1: Search strategies from the 4 databases, Supplementary Table S2: Supplementary search strategies from 2 databases, Supplementary Table S3: JBI Critical Appraisal Checklist for Case Series (Buongiorno et al. [19], 2023), Supplementary Table S4: Summarized findings.
