# Supplementary material for: Objective Sleep Architecture Alterations and Sleep-Dependent Brain Clearance Dysfunction Across the Early Alzheimer’s Disease Continuum: A Systematic Review
Source: J Clin Med. 2026 Aug 20;15(16):6454. doi: 10.3390/jcm15166454 (PMC13514128; doi:10.3390/jcm15166454)
Supplement: Supplementary file 1 [file jcm-15-06454-s001.zip › jcm-4491554-supplementary.pdf]

Supplementary Table S1. Search strategies from the 4 databases

Search Engine	Search Strategy	Search Date 09/06/2026 N
**PubMed**	((glymphatic[tiab] OR "glymphatic system"[tiab] OR ALPS[tiab] OR "DTI-ALPS"[tiab] OR "perivascular space"[tiab] OR "perivascular spaces"[tiab] OR "enlarged perivascular space"[tiab] OR "enlarged perivascular spaces"[tiab] OR aquaporin-4[tiab] OR AQP4[tiab]) AND ("Sleep"[Mesh] OR "Polysomnography"[Mesh] OR "sleep architecture"[tiab] OR "slow wave sleep"[tiab] OR "slow-wave sleep"[tiab] OR "slow wave activity"[tiab] OR "slow-wave activity"[tiab] OR SWA[tiab] OR REM[tiab] OR NREM[tiab] OR polysomnography[tiab] OR EEG[tiab] OR actigraphy[tiab] OR "sleep fragmentation"[tiab] OR "sleep efficiency"[tiab] OR "circadian rhythm"[tiab]) AND ("Alzheimer Disease"[Mesh] OR Alzheimer*[tiab] OR "Mild Cognitive Impairment"[Mesh] OR mild cognitive impairment[tiab] OR MCI[tiab]))	106
**Embase**	(glymphatic.ti,ab,kw.<br>OR glymphatic system.ti,ab,kw.<br>OR DTI-ALPS.ti,ab,kw.<br>OR "ALPS index".ti,ab,kw.)<br><br>AND<br><br>(sleep architecture.ti,ab,kw.<br>OR slow wave sleep.ti,ab,kw.<br>OR slow-wave sleep.ti,ab,kw.<br>OR slow wave activity.ti,ab,kw.<br>OR slow-wave activity.ti,ab,kw.<br>OR SWA.ti,ab,kw.<br>OR REM.ti,ab,kw.<br>OR NREM.ti,ab,kw.<br>OR polysomnography.ti,ab,kw.<br>OR EEG.ti,ab,kw.<br>OR actigraphy.ti,ab,kw.<br>OR sleep fragmentation.ti,ab,kw.<br>OR sleep efficiency.ti,ab,kw.<br>OR circadian rhythm.ti,ab,kw.)	108

	AND<br>(alzheimer*.ti,ab,kw.<br>OR mild cognitive impairment.ti,ab,kw.<br>OR MCI.ti,ab,kw.)	
**Cochrane**	#1 "Alzheimer Disease"[MeSH] OR "Alzheimer*"[tiab] OR "Mild Cognitive Impairment"[MeSH] OR "MCI"[tiab] OR "Preclinical Alzheimer*"[tiab] OR "Prodromal Alzheimer*"[tiab] OR "Early Alzheimer*"[tiab] OR "Subjective Cognitive Decline"[tiab] OR "SCD"[tiab]<br><br>#2 "Sleep"[MeSH] OR "Sleep Architecture"[tiab] OR "Polysomnography"[MeSH] OR "Polysomnogra*"[tiab] OR "Sleep Stages"[MeSH] OR "Slow-wave sleep"[tiab] OR "Non-REM"[tiab] OR "NREM"[tiab] OR "REM sleep"[tiab] OR "Sleep Spindles"[tiab] OR "K-complex"[tiab] OR "Sleep Fragmentation"[tiab] OR "Sleep Depth"[tiab] OR "EEG sleep"[tiab] OR "Actigraphy"[MeSH]<br><br>#3 "Glymphatic System"[MeSH] OR "Glymphatic"[tiab] OR "Brain Clearance"[tiab] OR ("Interstitial fluid"[tiab] AND "clearance"[tiab]) OR "Perivascular space*"[tiab] OR "Virchow-Robin space*"[tiab] OR ("CSF"[tiab] AND "dynamics"[tiab]) OR "Meningeal lymphatic*"[tiab] OR "Aquaporin-4"[tiab] OR "AQP4"[tiab] OR "Beta-amyloid clearance"[tiab] OR "Tau clearance"[tiab] OR "Intrathecal contrast"[tiab] OR ("Diffusion MRI"[tiab] AND "perivascular"[tiab])<br><br>#1 AND #2 AND #3	22
**PsycINFO**	#1 glymphatic.tw. OR "glymphatic system".tw.<br><br>#2 "sleep architecture".tw. OR "slow wave sleep".tw. OR "slow-wave sleep".tw. OR "slow wave activity".tw. OR "slow-wave activity".tw. OR SWA.tw. OR REM.tw. OR NREM.tw. OR polysomnography.tw. OR EEG.tw. OR actigraphy.tw. OR "sleep fragmentation".tw. OR "sleep efficiency".tw. OR "circadian rhythm".tw.<br><br>#3 alzheimer*.tw. OR "mild cognitive impairment".tw. OR MCI.tw.	17

	#1 AND #2 AND #3

Supplementary Table S2. Supplementary search strategies from 2 databases

Search Engine	Search Strategy	Search Date 16/06/2026 N
**PubMed**	("Glymphatic System"[Mesh]<br>OR glymphatic[tiab]<br>OR "glymphatic system"[tiab]<br>OR ALPS[tiab]<br>OR "DTI-ALPS"[tiab]<br>OR "ALPS index"[tiab]<br>OR "perivascular space"[tiab]<br>OR "perivascular spaces"[tiab]<br>OR "enlarged perivascular space"[tiab]<br>OR "enlarged perivascular spaces"[tiab]<br>OR PVS[tiab]<br>OR aquaporin-4[tiab]<br>OR AQP4[tiab]<br>OR "BOLD-CSF"[tiab]<br>OR "CSF dynamics"[tiab]<br>OR "cerebrospinal fluid dynamics"[tiab]<br>OR "cerebrospinal fluid flow"[tiab]<br>OR "brain clearance"[tiab]<br>OR "waste clearance"[tiab]<br>OR "amyloid clearance"[tiab]<br>OR "tau clearance"[tiab]<br>OR "meningeal lymphatic*"[tiab])<br>AND<br>("Sleep"[Mesh]<br>OR "Polysomnography"[Mesh]<br>OR "sleep architecture"[tiab]<br>OR "slow wave sleep"[tiab]<br>OR "slow-wave sleep"[tiab]<br>OR "slow wave activity"[tiab]<br>OR "slow-wave activity"[tiab]<br>OR SWA[tiab]<br>OR REM[tiab]	166

	OR NREM[tiab]<br>OR polysomnography[tiab]<br>OR EEG[tiab]<br>OR actigraphy[tiab]<br>OR "sleep fragmentation"[tiab]<br>OR "sleep efficiency"[tiab]<br>OR "circadian rhythm"[tiab]<br>OR "sleep spindle*"[tiab]<br>OR "slow oscillation*"[tiab]<br>OR "K-complex*"[tiab]<br>OR coupling[tiab])<br>AND<br>("Alzheimer Disease"[Mesh]<br>OR Alzheimer*[tiab]<br>OR "Mild Cognitive Impairment"[Mesh]<br>OR "mild cognitive impairment"[tiab]<br>OR MCI[tiab]<br>OR "subjective cognitive decline"[tiab]<br>OR SCD[tiab]<br>OR "preclinical Alzheimer*"[tiab]<br>OR "prodromal Alzheimer*"[tiab]<br>OR "early Alzheimer*"[tiab]<br>OR "amyloid positive"[tiab]<br>OR "amyloid-positive"[tiab]<br>OR "biomarker positive"[tiab]<br>OR "biomarker-positive"[tiab])	
**Embase**	(glymphatic.ti,ab,kw.<br>OR "glymphatic system".ti,ab,kw.<br>OR DTI-ALPS.ti,ab,kw.<br>OR ALPS.ti,ab,kw.<br>OR "ALPS index".ti,ab,kw.<br>OR "perivascular space*".ti,ab,kw.<br>OR PVS.ti,ab,kw.<br>OR aquaporin-4.ti,ab,kw.<br>OR AQP4.ti,ab,kw.<br>OR "BOLD-CSF".ti,ab,kw.<br>OR "CSF dynamics".ti,ab,kw.<br>OR "cerebrospinal fluid dynamics".ti,ab,kw.<br>OR "cerebrospinal fluid flow".ti,ab,kw.<br>OR "brain clearance".ti,ab,kw.<br>OR "waste clearance".ti,ab,kw.<br>OR "amyloid clearance".ti,ab,kw.	211

OR "tau clearance".ti,ab,kw.
OR "meningeal lymphatic*".ti,ab,kw.)

AND

(sleep architecture.ti,ab,kw.
OR "slow wave sleep".ti,ab,kw.
OR "slow-wave sleep".ti,ab,kw.
OR "slow wave activity".ti,ab,kw.
OR "slow-wave activity".ti,ab,kw.
OR SWA.ti,ab,kw.
OR REM.ti,ab,kw.
OR NREM.ti,ab,kw.
OR polysomnography.ti,ab,kw.
OR EEG.ti,ab,kw.
OR actigraphy.ti,ab,kw.
OR "sleep fragmentation".ti,ab,kw.
OR "sleep efficiency".ti,ab,kw.
OR "circadian rhythm".ti,ab,kw.
OR "sleep spindle*".ti,ab,kw.
OR "slow oscillation*".ti,ab,kw.
OR "K-complex*".ti,ab,kw.
OR coupling.ti,ab,kw.)

AND

(alzheimer*.ti,ab,kw.
OR "mild cognitive impairment".ti,ab,kw.
OR MCI.ti,ab,kw.
OR "subjective cognitive decline".ti,ab,kw.
OR SCD.ti,ab,kw.
OR "preclinical Alzheimer*".ti,ab,kw.
OR "prodromal Alzheimer*".ti,ab,kw.
OR "early Alzheimer*".ti,ab,kw.
OR "amyloid positive".ti,ab,kw.
OR "amyloid-positive".ti,ab,kw.
OR "biomarker positive".ti,ab,kw.
OR "biomarker-positive".ti,ab,kw.)

Supplementary Table S3. JBI Critical Appraisal Checklist for Case Series (Buongiorno et al. [19], 2023)

JBI Case Series Criterion	Assessment	Justification
**1. Were there clear criteria for inclusion in the case series?**	**Yes**	Participants were adults with mild-to-moderate cognitive impairment and CSF biomarker evidence of Alzheimer's disease undergoing serial intrathecal gadobutrol MRI.
**2. Was the condition measured in a standard, reliable way for all participants?**	**Yes**	Alzheimer's disease pathology was confirmed using CSF biomarkers, and all participants underwent the same diagnostic work-up.
**3. Were valid methods used for identification of the condition?**	**Yes**	Established biomarker-supported diagnostic criteria and standardized neuroimaging were used.
**4. Did the case series include consecutive participants?**	**Unclear**	The authors did not state whether participants were recruited consecutively.
**5. Did the case series have complete inclusion of participants?**	**Unclear**	It is unclear whether all eligible patients during the recruitment period were included.
**6. Was there clear reporting of participant demographics?**	**Yes**	Age, sex, cognitive status, biomarker findings and relevant clinical characteristics were reported.
**7. Was there clear reporting of clinical information?**	**Yes**	Sleep findings, cognitive impairment, biomarker status, MRI protocol and follow-up imaging were described in detail.
**8. Were the outcomes or follow-up results clearly reported?**	**Yes**	Serial MRI findings, sleep abnormalities, tracer retention and adverse events were clearly presented.
**9. Was there clear reporting of the presenting site(s)/clinic(s)?**	**Yes**	The recruiting centre and imaging procedures were adequately described.
**10. Was statistical analysis appropriate?**	**Not applicable**	This descriptive case series reported individual patient findings; no inferential statistical analyses were performed or required.

Supplementary Table S4. Summarized findings

Study	Country	Design	Population	N	Objective sleep assessment	Brain clearance measure	Main outcome
**Liu et al., 2026 [16]**	China	Prospective observational	Biomarker-positive AD + controls	75	PSG with EEG oscillatory coupling	DTI-ALPS, BOLD-CSF coupling, PVS, CP volume	Altered sleep oscillations associated with impaired glymphatic biomarkers

**Zheng et al., 2026 [17]**	China	Cross-sectional	Alzheimer's disease + controls	260	Overnight polysomnography	DTI-ALPS	Increasing OSA severity associated with lower ALPS index in AD
**Falgàs et al., 2026 [18]**	Spain	Cross-sectional	Controls, MCI, AD	58	Overnight polysomnography	MRI-visible PVS, locus coeruleus MRI, CSF noradrenaline	Reduced slow-wave sleep associated with greater PVS burden
**Buongiorno et al., 2023 [19]**	Spain	Case series	Mild-to-moderate AD	4	Polysomnography	Intrathecal gadobutrol MRI tracer	Delayed tracer clearance associated with abnormal sleep
